# Theta Oscillations at Subthalamic Region Predicts Hypomania State After Deep Brain Stimulation in Parkinson's Disease

**DOI:** 10.3389/fnhum.2021.797314

**Published:** 2021-12-20

**Authors:** Yi-Chieh Chen, Hau-Tieng Wu, Po-Hsun Tu, Chih-Hua Yeh, Tzu-Chi Liu, Mun-Chun Yeap, Yi-Ping Chao, Po-Lin Chen, Chin-Song Lu, Chiung-Chu Chen

**Affiliations:** ^1^Division of Movement Disorders, Department of Neurology, Chang Gung Memorial Hospital, Taoyuan, Taiwan; ^2^Neuroscience Research Center, Chang Gung Memorial Hospital, Taoyuan, Taiwan; ^3^College of Medicine, Chang Gung Memorial Hospital, Taoyuan, Taiwan; ^4^Department of Mathematics, Duke University, Durham, NC, United States; ^5^Department of Statistical Science, Duke University, Durham, NC, United States; ^6^Department of Neurosurgery, Chang Gung Memorial Hospital, Taoyuan, Taiwan; ^7^Department of Neuroradiology, Chang Gung Memorial Hospital, Taoyuan, Taiwan; ^8^Graduate Institute of Biomedical Engineering, Chang Gung University, Taoyuan, Taiwan; ^9^Professor Lu Neurological Clinic, Taoyuan, Taiwan

**Keywords:** Parkinson's disease, subthalamic deep brain stimulation, local field potentials (LFP), theta oscillation, hypomania

## Abstract

Subthalamic nucleus (STN) deep brain stimulation (DBS) is an effective treatment for the motor impairments of patients with advanced Parkinson's disease. However, mood or behavioral changes, such as mania, hypomania, and impulsive disorders, can occur postoperatively. It has been suggested that these symptoms are associated with the stimulation of the limbic subregion of the STN. Electrophysiological studies demonstrate that the low-frequency activities in ventral STN are modulated during emotional processing. In this study, we report 22 patients with Parkinson's disease who underwent STN DBS for treatment of motor impairment and presented stimulation-induced mood elevation during initial postoperative programming. The contact at which a euphoric state was elicited by stimulation was termed as the hypomania-inducing contact (HIC) and was further correlated with intraoperative local field potential recorded during the descending of DBS electrodes. The power of four frequency bands, namely, θ (4–7 Hz), α (7–10 Hz), β (13–35 Hz), and γ (40–60 Hz), were determined by a non-linear variation of the spectrogram using the concentration of frequency of time (conceFT). The depth of maximum θ power is located approximately 2 mm below HIC on average and has significant correlation with the location of contacts (*r* = 0.676, *p* < 0.001), even after partializing the effect of α and β, respectively (*r* = 0.474, *p* = 0.022; *r* = 0.461, *p* = 0.027). The occurrence of HIC was not associated with patient-specific characteristics such as age, gender, disease duration, motor or non-motor symptoms before the operation, or improvement after stimulation. Taken together, these data suggest that the location of maximum θ power is associated with the stimulation-induced hypomania and the prediction of θ power is frequency specific. Our results provide further information to refine targeting intraoperatively and select stimulation contacts in programming.

## Introduction

High-frequency deep brain stimulation (DBS) in the subthalamic nucleus (STN) area has been proven to be an effective treatment for patients with advanced Parkinson's disease (Limousin et al., 1995; Deep-Brain Stimulation for Parkinson's Disease Study Group et al., 2001; Chen et al., 2003; Krack et al., 2003). Nevertheless, pathological behaviors or changes in mood, such as mania, hypomania, impulsive or explosive behaviors, may be reported after STN DBS in patients with Parkinson's disease (Krack et al., 2001; Kulisevsky et al., 2002; Frank et al., 2007; Mallet et al., 2007). It has been suggested that the occurrence of these symptoms depends on the anatomical location of stimulating contacts in STN or its surrounding structures as substantia nigra or triangle of Sano (Bejjani et al., 1999, 2002). Numerous observations from animal studies and human functional magnetic resonance imaging (MRI) have suggested three functional subdivisions, namely, motor, cognitive, and emotional, in STN (Alexander et al., 1986; Parent and Hazrati, 1995; Mallet et al., 2007; Rossi et al., 2015). Therefore, the STN may play a crucial role in controlling complex behaviors including motor and non-motor components (Castrioto et al., 2014).

Electrophysiological recordings demonstrate that impulsive behavior in Parkinson's disease is associated with low-frequency activity in STN (Rodriguez-Oroz et al., 2011). Changes in STN α-θ activity occur in response to emotional processing (Kuhn et al., 2005; Burbaud et al., 2013; Eitan et al., 2013). The association between α-θ activity and ventral STN is further supported by single-unit recordings (Rappel et al., 2018, 2020) and connectivity studies that map local field potentials (LFP) to imaging (Accolla et al., 2016).

In this study, we test the hypothesis that intraoperative recording of LFP activities may predict the DBS contact that may cause behavioral changes when stimulated postoperatively. To do this, we introduced the DBS electrodes in steps that allowed LFPs to be recorded at different depths. The value of step recording is supported by previous studies (Chen et al., 2006; Yoshida et al., 2010; Milosevic et al., 2020). Intraoperative LFP recorded from DBS electrodes as they descend to the target provides information from β oscillations about the depth of the motor domain of the STN (Chen et al., 2006) and helps to predict the therapeutic efficacy of chronic stimulation for Parkinson's disease (Yoshida et al., 2010).

We retrospectively analyzed initial programming records performed 1 month after DBS electrode implantation. The hypomania-inducing contacts (HICs) during postoperative programming of the electrical parameters for therapeutic stimulation were defined and correlated with the LFP recorded intraoperatively in the stepped descent procedure. We report an electrophysiological biomarker, θ oscillations, that predicts the HICs. The relevance of this is that this predictive information might help intraoperative targeting and postoperative programming to avoid behavioral disturbance when treating motor impairments.

## Materials and Methods

### Patients and Surgery

A total of 70 patients with idiopathic Parkinson's disease who received bilateral STN DBS for the treatment of Parkinsonism between 2010 and 2020 in the Chang Gung Memorial Hospital were assessed. Patients participated with informed consent and the permission of the local ethics committee. Indications for surgery were advanced Parkinson's disease with motor fluctuations and/or dyskinesia. Preoperative assessments included the Unified Parkinson's Disease Rating Scale (UPDRS) on and off medication, Mini-Mental State Examination (MMSE), and Beck Depression Inventory.

Patients were operated on after overnight withdrawal of levodopa and dopamine agonists. The implantation of DBS electrodes was performed under local anesthesia. The electrode used was model 3389 (Medtronic Neurological Division, MN, USA) with four platinum-iridium cylindrical surfaces (1.27 mm diameter and 1.5 mm length), 1.5 mm per contact, and 0.5 mm per separation between each contact. Contacts 0 and 3 were the most ventral and dorsal, respectively. For all patients who were operated on after 2017, whole-brain (from vertex to foramen magnum) axial T1-weighted image (T1WI) and T2-weighted image (T2WI) 3D MRIs were routinely arranged for trajectory planning (O'Gorman et al., 2011; Chandran et al., 2016) except for 11 patients who were operated before 2017. A whole-brain stereotactic non-enhanced CT was also obtained after the application of the Cosman-Roberts-Wells (CRW) frame (Integra Radionics, Burlington, MA, USA). All sequences were performed using 1-mm slice thickness and in a continuous fashion. The images were transferred to the DICOM database using the StealthStation S7 navigation system (Medtronic, Neurological Division, MN, USA). The image fusion software fused the two sets of MRI and stereotactic CT images to form a 3D reconstruction. The trajectories were aimed at the center of STN, by direct visualization on T2-weighted axial, coronal, and sagittal MRI. These images were superimposed on stereotactic CT to define the area corresponding in location to the STN in the atlas of Schaltenbrand and Wahren (Schaltenbrand and Wahren, 1977). For the 11 patients without preoperative 3D MRI, the trajectories were planned using the indirect calculation method (Tu et al., 2018). The preliminary indirect targets were calculated relative to the anterior commissure (AC), posterior commissure (PC), and mid-commissural point (MCP) on stereotactic CT images. The indirect target coordinates were calculated to target the STN region 12 mm lateral from the AC-PC line, 3 mm posterior to the MCP, and 4–5 mm inferior to the MCP (Tu et al., 2018). Intraoperative microelectrode recording (MER) was performed to guide the targeting. The level of entry and exit of STN and substantia nigra reticulata (SNr) were determined using MER. After the microelectrode was withdrawn, the DBS electrode was advanced in steps of 1 mm from a point 1–2 mm above the MER defined “entry” to STN to a point 2 mm below the “exit” of STN. The final targeting for stimulation electrode implantation was selected according to the results of MER. Intraoperative bipolar stimulation was performed using an external stimulator (Medtronic dual screen 3625, MN, USA) to test the clinical effect and any side effects once the DBS electrode completed its trajectory. Postoperative whole-brain MRI in a 1.5T scanner was routinely arranged before pulse generator implantation. A fast MRI scan with 2D T1WI and T2WI parallel to the DBS leads was performed. Whole-brain 3D TIWI and T2WI were acquired in the axial plane and reconstructed in oblique-sagittal and oblique-coronal fashion along the DBS leads. DBS leads position reconstruction was performed using MATLAB-based software (Lead-DBS, RRID:SCR_002915) (Horn and Kuhn, 2015; Horn et al., 2019) in 11 patients who had both preoperative and postoperative 3D MRI. Final 3D rendering of the DBS leads was demonstrated relative to the sensorimotor, associative, and limbic subregions of STN defined using the DISTAL Atlas (Ewert et al., 2018).

### Stimulation Programming

The initial programming was arranged 1 month after the surgery to minimize the possible stun effects, and dopaminergic medications were withdrawn for at least 12 h. Two experienced movement disorder specialists (C.C.C. and Y.C.C.) assessed systematically. While stimulation parameters were set at the frequency of 130 Hz and the pulse width of 60 us, the voltage was progressively increased with a fixed step of 0.2 or 0.5 V up to 3.5 V. Rigidity of the contralateral wrist was measured as the objective primary motor improvement with stimulation (Moro et al., 2002). Tremor and/or bradykinesia were also evaluated. The voltage thresholds for motor control and adverse effects, such as contraction, sensory abnormality, dysarthria, gaze palsy and diplopia, and dizziness at each of the four contacts on each electrode, were determined. All patients were set to monopolar stimulation, and the contact with the greatest improvement in motor symptoms and least adverse effects was selected for chronic stimulation.

We particularly assessed the occurrence of stimulation-induced hypomania. The definition of hypomania state was referred from *the Diagnostic and Statistical Manual of Mental Disorders (DSM-5)* (American Psychiatric Association, 2013), except for the disease duration. Stimulation-induced hypomania occurred within 1 min after stimulation. The presentation included euphoria, expansive laughing, flight of ideas, hyperactivity, and elation. Most patients did not have emotional insight, and the unusualness was observed by the doctors and caregivers. Only three patients stated their inner urge of acting out. The hypomania state disappeared soon after the DBS was turned off, while the motor improvement lasted longer. If the hypomania state could be provoked at more than one contact, only the one with the lower/lowest voltage threshold was termed as the HIC and selected for further analysis. The depth of the lower margin of the HIC was described with reference to the planned surgical target.

### Intraoperative LFP Recordings

Intraoperative LFP recordings were made when the DBS electrode descended with steps of 1 mm. The patients were awake and resting. The LFP at each step was recorded for at least ~60 s, except for the last step at which ~200 s of recordings were made (range from 60.38 to 229.63 s, 81.87 ± 2.16 s). LFPs were recorded unipolarly from the four adjacent contacts of each DBS electrode and subjected to a common average reference. The signals were amplified, band filtered between 1 and 128 Hz, and sampled at 2,048 Hz (Porti Amplifier; TMSI international, Enschede, the Netherlands). Purpose written software saved the original time series on a portable laptop that displayed online the evolving patterns of β band power from each contact as the DBS electrode was descending (written by Pogosyan A). We selected the signal recording from contact 0 and 1 for further signal processing to avoid the stun effect of the immediate tissue damage by the electrodes. LFPs from 34 sides were excluded due to technical errors during recordings. Therefore, only 106 sides (24 HICs and 82 non-HICs) were included for further evaluation.

### Signal Processing Using ConceFT

The LFP recordings were transformed offline in Spike 2 (Cambridge electronic design, RRID:SCR_000903) to work in MATLAB R2017b software (Mathworks, Natick, MA, USA, RRID:SCR_001622). The bipolar signal between contacts 0 and 1 was calculated offline and analyzed. The first 60-s signal in every recording was taken for analysis. In preprocessing, locally weighted scatterplot smoothing was done to remove the trend of LFP. The signals were downsampled to 200 Hz. The signals ranging from 5 to 55 Hz and 65 to 95 Hz were evaluated by the root mean square (RMS), and the whole signal was divided by the RMS to normalize the data (Lofredi et al., 2019). The normalized power spectrum was obtained using the concentration of frequency and time (conceFT) (Daubechies et al., 2016). In brief, the algorithm proceeds as follows:

1. The top four Hermite functions restricted to [−6, 6] s and contracted to [−2, 2] s were selected. They were uniformly and independently combined linearly thirty times to produce thirty windows.
2. For each window, the synchrosqueezing transform (SST) of each LFP was evaluated with the window as the kernel and with 90% overlapping to obtain a time-frequency representation (TFR) with a resolution of ~0.2 Hz in the frequency domain and 50 ms in the time domain.
3. The time-varying power spectrum (tvPS) of each LFP was obtained by averaging all TFRs determined by SST. This step is a generalized multitaper technique (Donald and Walden, 1993) called conceFT.
4. The overall energy of the LFP over the frequency band of interest was obtained by summing the energy of the tvPS over that frequency band.

**Figure 1:**
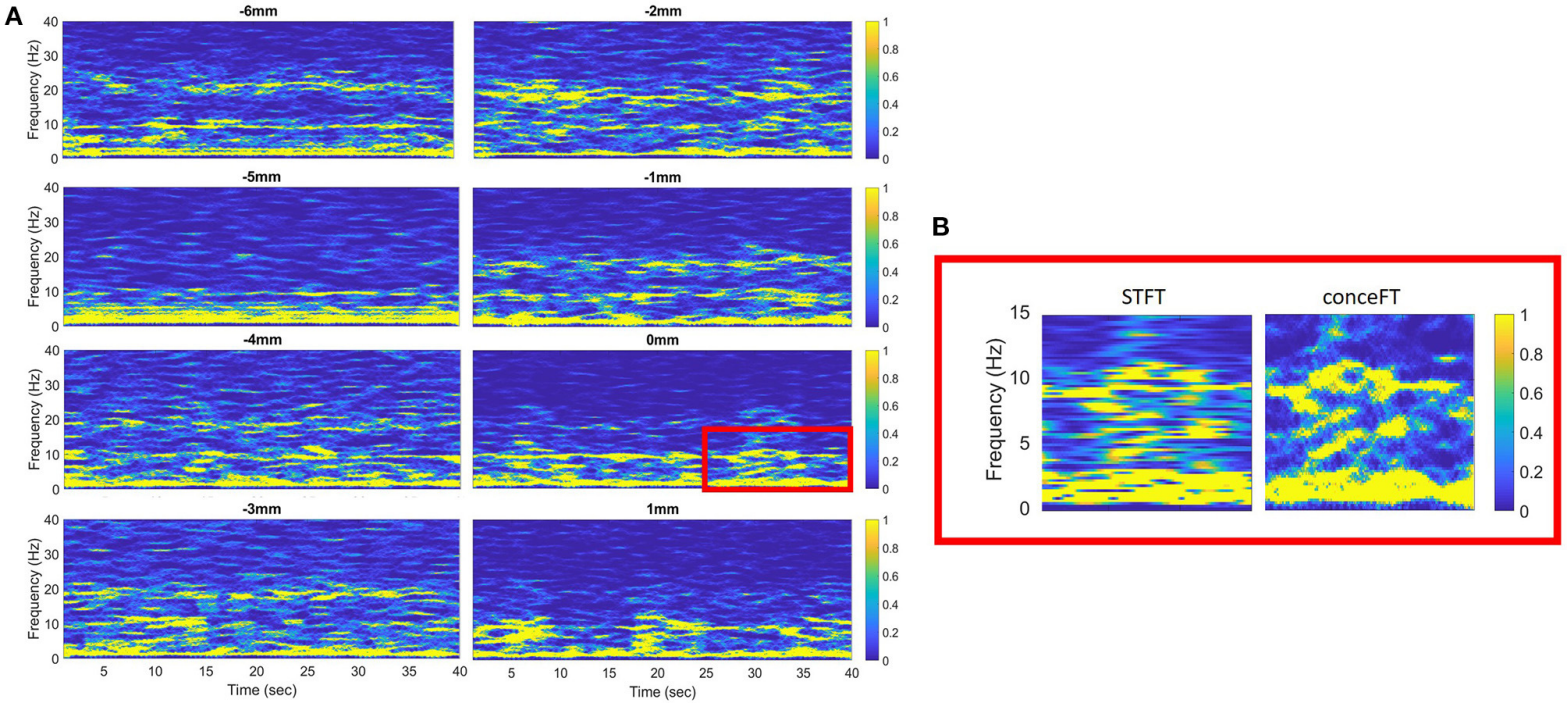
**(A)** The time-frequency representation of each descending step of the macroelectrode was analyzed using the concentration of frequency of time (conceFT) in patient 22. The signal is bipolar, from contact 0 and contact 1. Negative and positive distances represent depth above and below the surgical target, respectively. **(B)** The zoom-in picture of the red box in **(A)**. It shows the different resolutions of the conceFT and traditional spectrogram determined using short-time Fourier transform (STFT), where the Gaussian function, the first Hermite function, is used in STFT, and the window lengths in both conceFT and STFT are the same (Lin and Wu, 2017).

In previous studies, we used either the change of power or peak power to define the local generators of different frequency bands. However, it is well-known that the resolution of traditional short-time Fourier transform (STFT) in differentiating narrow band activities is limited (Wu, 2020). We demonstrated the advantage of conceFT in Figure 1B. Accordingly, we used conceFT to measure the spectral power as this technique can sharpen the spectrum at each moment, capture the non-stationary dynamics, and stabilize the impact of noise (Daubechies et al., 2016; Lin and Wu, 2017; Wu and Liu, 2018). LFP power was determined in four candidate frequency bands, namely, θ (4–7 Hz), α (7–10 Hz), β (13–35 Hz), and γ (40–60 Hz), for every 1 mm point. Our algorithm automatically selected the maximum power in each candidate frequency band. The depth of the maximum power at each frequency band within the trajectory was selected for further analysis.

### Statistical Analysis

Descriptive statistics were used to identify the mean depth of the HIC and the depth of the maximum power in each frequency band. The distances between the location of the bottom of contacts and each frequency band maximum were calculated and presented as mean ± standard error. The associations between the depth of the HIC and electrophysiological features were assessed using Spearman's rank correlation. Partial correlation was performed to exclude confounding effects. It was selected since different frequency bands might be dependent and this dependence might impact the association between the frequency bands and the HIC depths and needs to be controlled. The normality of each parameter was confirmed with the Kolmogorov-Smirnov test, and for those parameters that did not pass the test, we applied the Box-Cox transform before running the partial correlation. To compare groups with and without HIC, we used Levene's test to assess the equality of variances and *t*-test to check the difference between the two groups. A *p*-value < 0.05 indicated statistical significance. Results from descriptive statistics were presented as mean ± standard error unless otherwise stated. All statistical analyses were performed using SPSS (Version 24, SPSS Inc., Chicago, IL, USA, RRID:SCR_019096).

## Results

In this retrospective study, HICs were noted on 24 sides of 22 patients. Within these 24 sides, the average span of STN determined using MER was 5.26 ± 0.20 mm. The postoperative improvement of the UPDRS III score was 48% ($\frac{OFF\;stimulation-ON\;stimulation}{OFF\;stimulation}\times 100\%$) after overnight withdrawal of dopaminergic medication. On the 24 sides on which HICs were noted, 11 HICs were one or two contacts below the chronic stimulation contacts, while 13 HICs were concordant with the active contact selected for chronic stimulation. On average, the chronic stimulation contacts were located 1.16 ± 0.29 mm above the HICs. Within these 24 contacts, 13 HICs located on the left and 11 HICs located in the right STN area. The characteristics of the 22 patients with HIC are presented in Table 1.

**Table 1:** Patient characteristics.

**No**.	**Age (year)/Sex**	**DD (year)**	**Pre-OP UPDRS III (On/Off)**	**Pre-OP manifestation**		UPDRS III (ON/OFF DBS in off drugs)^*^	**Hypomania-inducing contact**			Chronic stimulation contact (V)^***^
				**Motor symptoms**	**Psychiatric symptoms**		**Location**	Threshold (V)^**^	**Presentations**	
1	47/M	31	16/29	AR	–	27/54	C2	2.2	Elevated mood	C3/C10 (3.6/2.2)
2	62/M	14	27/50	Tremor	–	25/43	C8	3	Smile	C3/C10 (3.4/3.4)
3	52/M	14	20/42.5	AR	DDS, ICD	17/34	C0	1.5	Laughter	C2/C10 (3.3/3.2)
4	65/M	6	32/46	AR	Anxiety, depression	NA	C2	3	Smile	C3/C11 (3.1/3.1)
5	69/F	10	15/31	Tremor	–	12/36	C2	1.6	Elevated mood, smile	C2/C10 (3.7/3.7)
6	53/F	26	23.5/50	Tremor	–	45/79	C10	3	Stand up and rush to walk	C1/C11 (3.6/3.7)
7	52/F	15	18/32.5	AR	–	13/39	C3	2	Elevated mood	C3/C8 (3.5/3.5)
8	65/M	18	34/38	Tremor	Depression	27/77.5	C10	3	Smile	C2/C10 (3.6/3.6)
9	72/F	9	16/30	AR	Hallucination	35/53	C2	3	Burst into laughter	C2/C10 (3.5/3.5)
10	64/F	19	20/33	Tremor	–	28/43	C1/C11	2/2.7	Burst into laughter	C2/C11 (3.2/3.4)
11	63/M	25	13/28	Tremor	–	15/36	C11	3	Smile	C1/C11 (3.7/3.7)
12	64/F	16	28/51	Tremor	ICD, punding	43/64.5	C1	3	Agitation, Violent behaviors	C1/C11 (3.3/3.7)
13	62/F	9	11/28	AR	VH	16/37	C1	2.5	Laughter	C2/C11 (3.2/3.4)
14	75/F	7	15/58	AR	–	33/49	C10	2	Smile	C2-3/C10-11 (3.7/3.7)
15	63/F	21	29/48	AR	VH, depression	28/65	C2	1	Burst into laughter	C2/C10 (3.3/3.6)
16	65/M	10	22/67	Tremor	VH	34/51	C10	2.2	Talk about his private things	C2/C11 (3.5/3.5)
17	72/M	17	24/33	Tremor	ICD	18/31	C11	0.5	Invite doctors to a meal, display his collection of ancient coins	C3/C11 (3.3/3.3)
18	69/F	12	17/29	Tremor	–	NA	C2	2	Burst into laughter	C3/C11 (2.7/1.8)
19	57/M	15	9/30	AR	–	12/41	C11	2	Sexual teasing	C1/C11 (2.9/3.0)
20	51/M	14	25/52	AR	ICD, DDS	13/23	C1	2	Burst into laughter	C3/C10 (3.5/3.5)
21	73/F	14	30/50	AR	VH, depression	17/47	C11	3	Compulsive talking, Sing in the lab	C2/C11 (3.5/3.4)
22	60/M	10	25/41	Tremor	- -	23/43	C2/C9	1.8/1.7	Show off the stock trading records, demonstrate running upstairs	C2/C10 (2.8/2.8)

*AR, akinesia-rigidity; C, contact; DD, disease duration; DDS, dopamine dysregulation syndrome; F, female; ICD, impulse control disorder; OP, operation; M, male; V, voltage; VH, visual hallucination; UPDRS, Unified Parkinson's Disease Rating Scale*.

* *The UPDRS here presents the current status of patients, 11 of them received operation for over than 5 years*.

** *All with 130 Hz, 60 us*.

*** *The final chosen contacts after first programming and in the outpatient department*.

### Characteristics of the LFP

An example of the offline analysis of LFP activity from the lowermost contact pair (C01) recorded during successive descending steps of the macroelectrode on the right side of the case 22 is illustrated in Figure 1A. The depths with maximum power for each frequency band were selected for further analysis.

### Postoperative Contact Localization

**Figure 2:**
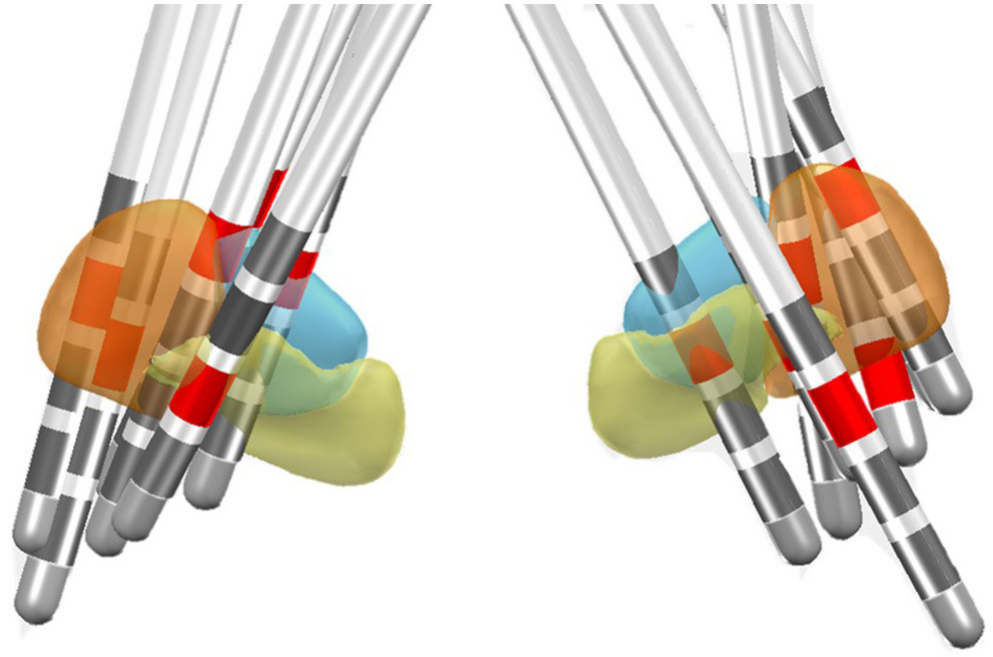
Location of the HIC in the DISTAL functional atlas in MNI space for 13 electrodes in 11 patients (Ewert et al., 2018). The sensorimotor subregion of the STN is represented in the orange, associative subregion in blue, and the limbic subregion in yellow. The HICs are marked in red.

Among these 22 patients who had HICs, postoperative MRI reconstruction was only available in 11 patients who had both preoperative and postoperative 3D MRI. Among these 11 patients, 12 HICs were detected. The reconstructions showed that two HICs involved the motor subregion, one passed through the associative subregion, two involved the border between the motor and associate subregion, two involved the border between the motor and limbic subregion, and one contact was thought to lie at the border of the associative and limbic subregions. Four contacts did not touch STN. An illustration of the 13 HICs is shown in Figure 2.

### The Relative Distance Between the Depths of the HIC and Maximum Power at Different Frequency Bands

**Figure 3:**
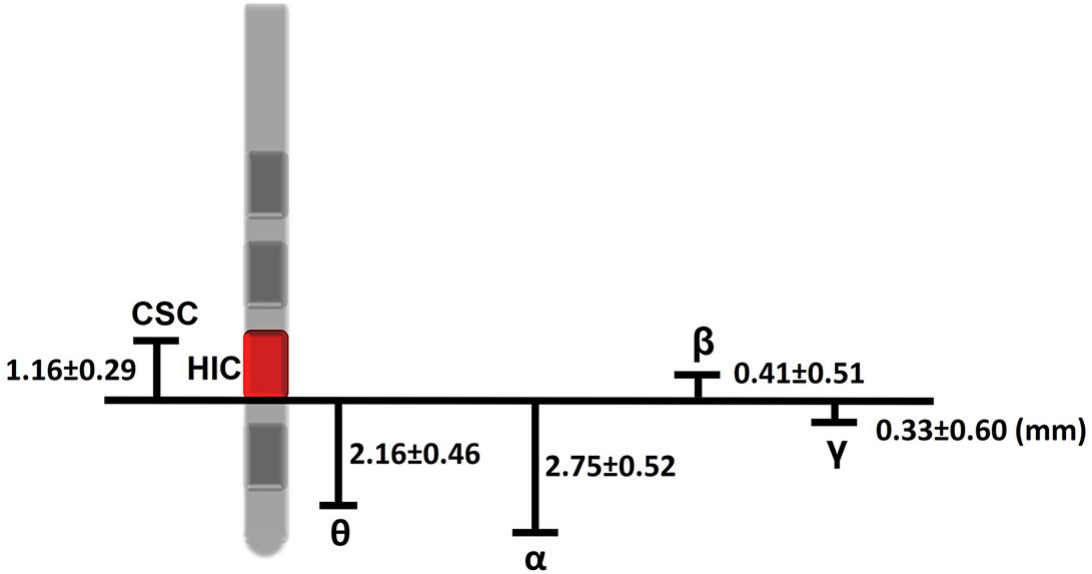
Schematic of the distances involved between the intraoperatively and postoperatively defined levels. The HIC in this example is contact 1 and is shown in red. Measurements given in the text and to this figure are with respect to the lower margin of this contact. Accordingly, we can estimate that the chronic stimulation contact was above HIC about 1.16 mm, and the HIC was about 0.41 mm below the maximum β power. 2.16, 2.75, and 0.33 mm above the levels of maximum θ, α, and γ, respectively. Chronic stimulation contact (CSC) located 1.16 mm above HIC. Estimates of the distances involved were drawn from the mean difference between different depths (presented as mean ± standard error).

On average, the depth of maximum θ power was 2.16 ± 0.46 mm below that of the HIC, while the depth of maximum β power was 0.41 ± 0.51 mm above that of the HIC (Figure 3). The depths of maximum α and γ power were 2.75 ± 0.52 mm and 0.33 ± 0.60 mm below that of the HIC, respectively. The mean difference of the depths of maximum θ and α, θ, and β, and α and β power were 0.59, 2.57, and 3.16 mm, respectively, which indicated that maximum θ and α power were located ~2 and ~3 mm below the maximum β power, respectively. All levels were taken as the deepest margin of the respective contact (Figure 3).

### The Correlation Between the Depth of the HIC and Maximum Power at Different Frequency Bands

We evaluated the correlation between the level with the maximum LFP power at contact pair C01 in each of the four frequency bands and the level of the HIC using Spearman's rank correlation. There were strong correlations between the depth of maximum power in the θ (*r* = 0.676, *p* < 0.001) and β frequency bands (*r* = 0.618, *p* = 0.001) determined intraoperatively and the depth of the HIC. In addition, there was a significant but slightly weaker correlation between the HIC and the level of maximum α power (*r* = 0.494, *p* = 0.014). There was no significant correlation for the depth of maximum γ band (*r* = 0.296, *p* = 0.16). To exclude the cofounding interaction between θ, α, and β bands, a partial correlation was conducted. The correlation between the depth of maximum θ power and the HIC remained significant after partializing out the effect of the depths of maximum β (*r* = 0.474, *p* = 0.022) and maximum α (*r* = 0.461, *p* = 0.027) power. In contrast, the correlation between the depth of the maximum α or β power and HIC was no longer significant after partializing out the effect of the depth of the maximum θ power (*r* = 0.071, *p* = 0.747 and *r* = −0.026, *p* = 0.907, respectively). The results of the partial correlation are summarized in Table 2. The above partial correlation analysis suggests that the depth of maximum θ power encodes extra predictive information regarding the depth of the HIC over and above that afforded by the depth of maximal α or β power.

**Table 2:** Results of the partial correlation after adjustment with controlled variant.

		**HIC**		
	**Controlled variant**	**θ**	**α**	**β**
θ	*r*	–	0.461	0.474
	*p*	–	0.027^*^	0.022^*^
α	*r*	0.071	–	0.232
	*p*	0.747	–	0.287
β	*r*	−0.026	0.185	–
	*p*	0.907	0.398	–

*HIC, hypomania-inducing contact; r, coefficient of partial correlation*.

* *p < 0.05*.

### Comparison of θ Power in Sides With and Without HIC

Among the 44 sides in 22 patients, HIC was only noted in 24 sides. The maximum β and θ power recorded from these sides were compared with the maximum β and θ power from 82 sides in which no HICs were detected, respectively. Levene's test showed no significant difference in the equality of variances, and a *t*-test was used. There was no significant difference in maximum β power between the two groups (3.49 ± 0.04 and 3.55 ± 0.02, *p* = 0.097). The average maximum θ power in sides with HICs was significantly higher than the average maximum θ power recorded from sides without HICs (2.99 ± 0.08 and 2.63 ± 0.04, *p* < 0.001). To identify a possible contribution of baseline clinical features to the occurrence of stimulation-induced hypomania, we compared the age, gender, disease duration, medication, motor, and non-motor symptoms before operation between patients with HIC and those without HIC. However, no significant differences were found between these two groups (Table 3).

**Table 3:** Clinical characteristics of patients, UPDRS score, and differences between groups.

	**Patients with HIC** **(*n* = 22)**	**Patients without HIC** **(*n* = 32)**
Sex (women/men)	11/11	12/20
Mean age at operation	62.5 ± 7.8	63.1 ± 8.7
Mean disease duration	15.1 ± 1.4	13.8 ± 0.8
Non-motor symptoms before surgery		
ICD (y/n)	4/18	3/32
Depression (y/n)	7/15	11/21
MMSE	28.6 ± 0.4	28.5 ± 0.3
LEDD (mg)	1385.1 ± 78.9	1222.1 ± 85.0
UPDRS III		
Before surgery		
Off med	40.8 ± 2.4	42.2 ± 2.3
On med	21.3 ± 1.5	20.1 ± 1.5
After surgery		
Off med/Off stim	46.4 ± 3.3	51.6 ± 3.6
Off med/On stim	24.1 ± 2.2	28.5 ± 2.0
Improvement of UPDRS III (%)	47.70%	43.30%
Voltage	3.3 ± 0.1	3.3 ± 0.5

*Age and duration are expressed in years and data are presented as mean ± SE*.

*ICD, impulse control disorder; MMSE, Mini-Mental State Examination; LED, levodopa equivalent dosage*.

**Figure 4:**
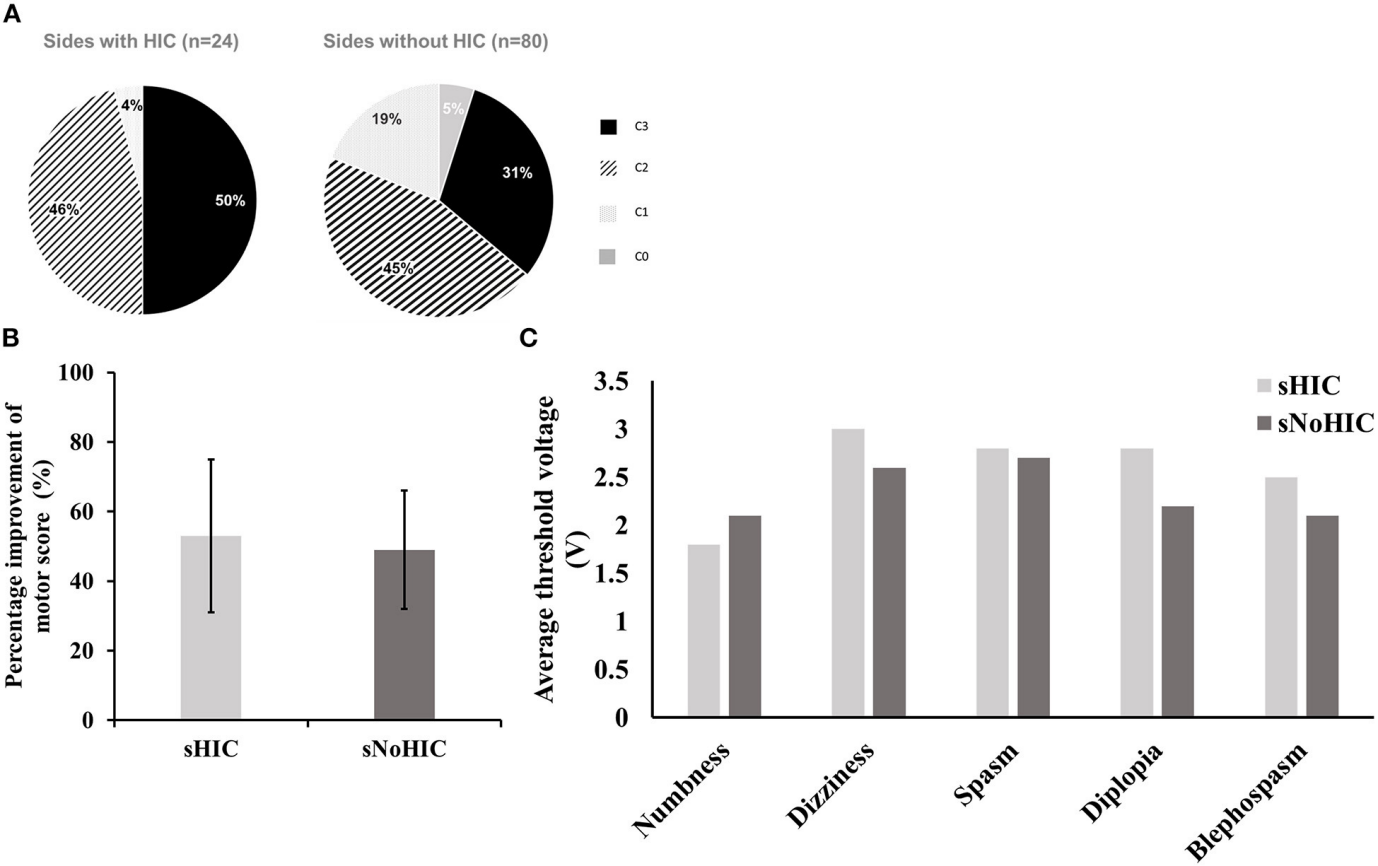
Comparison between sides with HIC (sHIC, *n* = 24) and those without HIC (sNoHIC, *n* = 80). **(A)** The distribution of chronic stimulation contacts in both groups (96 and 76% of chronic stimulation contacts located at dorsal contacts (C2 or C3) in sHIC and sNoHIC, respectively). **(B)** A bar graph presents the percentage of improvement of motor scores (UPDRS III sections 20–26) on the contralateral sides of sHIC and sNoHIC. Results were presented as mean ± SE. No significant difference was found between the two groups. **(C)** A similar bar graph presents the average threshold of side effects on each contact of each electrode between sHIC and sNoHIC. There were no significant differences between the two groups.

To further analyze these data, we divided 106 sides from 22 patients with HIC and 32 patients without HIC into two subgroups: sides with HIC (sHIC, *n* = 24), sides without HIC (sNoHIC, *n* = 80). Two sides were excluded due to incomplete data. Chronic stimulation contacts on sHIC tended to be more prevalent at dorsal contacts (C2 or C3) than on sNoHIC (96 vs. 76% in sHIC and sNoHIC, respectively, Figure 4A). We found no significant difference between the stimulation voltage in these two groups (3.3 ± 0.1 vs. 3.3 ± 0.5; *p* = 0.79). To compare the clinical response to DBS, contralateral UPDRS III subscores were assessed (sections 20–26) in two scenarios: off medication, off stimulation (OFF) and off medication, on stimulation (ON). No significant differences were found between the percentage of improvement of contralateral motor scores in these two groups (53.1 ± 4.7% vs. 50.0 ±2.2%, Figure 4B). The average threshold of side effects on each contact of each electrode was also found no significant difference between the two groups (Figure 4C).

## Discussion

We elicited HICs in 22 out of 70 patients with Parkinson's disease who underwent STN DBS surgery. HICs were identified on 24 sides with 54% on the left side and 46% on the right side. There was no significant difference in gender, age, disease duration, medication, motor, or non-motor symptoms before operation between patients with HIC or without HIC. Also, the motor improvements after DBS surgery were similar in the two groups, suggesting it was difficult to predict stimulation-induced hypomania across patients. We demonstrated that the depth of the maximum θ power determined using intraoperative recordings and the depth of the HICs selected during postoperative programming were strongly correlated. In addition, there was a weaker correlation between the depth of maximum α and β power recorded using intraoperative LFP recordings and the depth of the HICs. However, partial correlation confirmed that at least part of the predictive value of the depth of the maximum θ activity was independent of the depth of maximum α and β activity power. Conversely, partial correlation indicated that neither the depth of the maximum α nor the β activities were predictive of the HIC depth if the depth of the maximum θ power was considered. The correlation between θ oscillations and hypomania was further supported by our results that the sides with HICs had greater maximum θ power than those without HICs. Contrary to a previous report that demonstrated the lateralization of mood processing in the right STN (Eitan et al., 2013), our results showed that the HICs were equally distributed in both hemispheres.

Thus, the predictive value of maximum LFP power was driven by the θ band. This information may help refine targeting intraoperatively and the selection of stimulation contact during subsequent programming.

### The Utility of Intraoperative Stepping LFP Recordings

Why should we utilize intraoperative recording of macroelectrode steps rather than record LFP after electrodes are implanted? First, the stepped electrode descent helps ensure that the electrode is centered at the correct depth. Second, the spatial localization of oscillatory activities recorded from fixed electrodes is more limited, given neuronal signals can only be sampled from three contact pairs that are 2 mm apart. During the stepping procedure, neuronal activities within the STN area are recorded in descending steps of 1 mm so that spatial resolution is higher and the ambiguity in signal attribution with fixed bipolar recordings is avoided. Consider, for example, if electrode contacts 0 and 1 flank the generator of oscillatory activities, then, minimal power will be registered in the fixed bipolar montage due to signal cancellation. By stepping the electrode down at 1 mm increments, a point will come when the lowermost contact, C0, sits in or near the generator while contact C1 is still above it, giving a large potential gradient. The ambiguity inherent in fixed bipolar contacts can be circumvented by monopolar recordings, but these are more likely to saturate and the depths that are available for analysis are currently limited to four. This later limitation may be circumvented by next-generation DBS electrodes with a larger number of contacts separated in depth by less than 2 mm.

### The Electrophysiological Evidence for Functional Subregions of the STN and the Relative Location of Chronic Stimulation Contact, HIC, β, and θ Activity

In contrast to β oscillations that are dominant in the dorsolateral “motor” STN (Hammond et al., 2007; Zaidel et al., 2009, 2010), single-unit recordings in patients with Parkinson's disease suggest that θ-α oscillations are maximal in the ventromedial STN, thought to be related to the limbic subdivision (Rappel et al., 2018, 2020). Consistent with this, postoperative bipolar recording using DBS electrodes also suggests a different spatial distribution of β and α bands within the STN in Parkinson's disease (Rodriguez-Oroz et al., 2011; Accolla et al., 2016; Horn et al., 2017).

Although the depth of maximum θ power correlated with the depth of the HIC, the center of HIC was ~2 mm above the center of maximum θ power and much closer to the depth of maximum β activity. The most parsimonious explanation for this is that in addition to their “anti-kinetic” role in motor circuits (Kuhn et al., 2006; Hammond et al., 2007; Ray et al., 2008; Yoshida et al., 2010; Zaidel et al., 2010), β oscillations might be involved in cognitive or emotional control (Williams et al., 2003; Kuhn et al., 2004; Ray et al., 2009; Alegre et al., 2013). In line with this, recent studies reported emotional arousal and behavioral change with DBS stimulation in the motor part of STN (Irmen et al., 2019; Serranova et al., 2019). However, against this HICs were 1 or 2 contacts below the chronic stimulation contact in almost half of our patients.

Still, why is the HIC ~2 mm above the maximum θ activity? The primate studies show that the three motor, cognitive, and emotional domains in the STN have no sharply defined boundaries; instead, changes in functional localization are gradual (Mallet et al., 2007; Haynes and Haber, 2013). This is supported by our postoperative MRI contact reconstruction of 11 stimulation electrodes. While six of our HICs bordered the associate-limbic area, six HICs bordered the sensorimotor territory. The diffusion of stimulation is likely involving the limbic subregions. However, the proximity of contacts to the subregions of STN should only be considered as supportive evidence instead of proof of targeting, due to factors such as variability of structures and electrode artifact.

Another possible explanation of the discrepancy in depth between maximum θ oscillations and the HIC is perioperative brain shift (Miyagi et al., 2007; Halpern et al., 2008). The longer duration of stepped LFP recording procedure after prolonged MER recordings may aggravate the extent of brain shift. In addition, the procedure of anchoring the electrode and the snapping on of the caps that protect electrodes may push the DBS electrodes downward. This hypothesis needs to be tested by further investigations that contrast the LFPs recorded intraoperatively and a few days after the implantation.

It should be stressed that we have not assumed that the level of maximum θ power is within the limbic subdivision of STN. Although Mallet et al. (2007) demonstrated that stimulating at contacts in limbic STN induced mania, other studies suggested that mania was associated with stimulation of the substantia nigra pars reticulata (Kulisevsky et al., 2002; Ulla et al., 2011) or hypothalamus (Bejjani et al., 2002). Nevertheless, the fact that the average depth of maximum θ power was ~2 mm below the maximum β power suggests that the level of maximum θ power recorded intraoperatively is within the ventral domain of STN. Thus, the euphoric state noted in our patients could be attributed to direct stimulation at the associate-limbic territory of STN. This was further supported by our results showing that active contacts were more prevalent on contact 2 or 3 in sides with HIC than sides without HIC. A previous study had suggested that the active contacts lied within the motor domain of STN (Zaidel et al., 2010). If the dorsal contacts are located at the motor area in sHIC, then the ventral contact would likely lie in the limbic domain. Stimulation in this area would easily trigger hypomania.

### The Pathophysiology of θ and α Activity

The euphoric state observed in our patients is a complex behavior, characterized by mood elevation, disinhibition, and hyperactivity. Correlative data show that STN plays a crucial role in response inhibition (Baunez and Robbins, 1997; Frank et al., 2007; Eagle et al., 2008; Zavala et al., 2014, 2016). The power of θ activity in STN and the synchrony between θ oscillations in STN and medial prefrontal cortex (mPFC) activity increase in trials with high-conflict tasks (Cavanagh et al., 2011; Zavala et al., 2014, 2018). High-frequency stimulation suppresses θ oscillations in STN, disrupts mPFC-STN synchrony (Cavanagh et al., 2011), releases the brake on motoric/emotional processes, and may ultimately lead to neuropsychiatric behaviors (Halbig et al., 2009). It is noteworthy that in our series some patients reacted rashly with an urge to walk as soon as the HIC was stimulated. This observation is consistent with the computational “hold your horses” model of the effect of STN DBS (Frank et al., 2007).

It is also likely that α activity was related to the elevated mood changes. Maximal α and θ power were only ~1 mm apart. Electrophysiological recordings have suggested that α activity or reactivity in ventral STN correlates with the severity of depression (Huebl et al., 2011, 2014; Rappel et al., 2020), and an LFP-magnetoencephalography connectivity study showed that α activity in the STN was coherent with ipsilateral superior and contralateral inferior medial temporal cortex (Litvak et al., 2011), while θ oscillations were coherent with anterior cingulate cortex activity (Wojtecki et al., 2017).

In this study, we demonstrated that the patients with HIC tended to have higher θ activities which is partially dependent on the surgical trajectories. If the trajectory traversed the θ generator, then the proximity of the active electrode contact to this generator would trigger hypomania. However, it is probably also dependent on the pathophysiology in patients, that those who had higher θ activity were more susceptible to have mood change elicited by stimulation. This was consistent with a previous report that demonstrated the association between patients with PD with neuropsychiatric symptoms with 4–7.5 Hz activities (Rodriguez-Oroz et al., 2011).

### Limitations and Further Clinical Applications

Ultimately, the findings presented here only suggest that the depth of maximal θ activity may be an intraoperative biomarker of associate-limbic STN and that it might help predict the contact that is susceptible to mood/psychiatric disturbance when stimulated. However, further LFP-anatomical correlational studies are necessary to establish the former, and it remains to be seen whether HICs themselves predict the contact giving mood/psychiatric disturbance during chronic stimulation. Related to the latter, HICs were only found in about 30% of patients and so it remains to be seen whether the other 70% of patients are spared mood/psychiatric disturbances or not.

Notably, we only recorded LFP at different depths along a single trajectory, as the safety of multiple trajectories made by DBS electrodes remains uncertain (Seijo et al., 2014; Bjerknes et al., 2018). Information about neuronal activities across anterior-posterior dimensions is limited. Related to this, θ-α oscillations are only detected in <20% of neurons in multiple trajectories microelectrode recordings (Rappel et al., 2020), even though the latter recording technique affords a much better spatial resolution than LFP recorded by DBS electrodes. Notably, we only test the change in the motor but not acute emotional symptoms during the intraoperative macrostimulation due to the time restraint of DBS implantation.

In addition, HICs were defined during the standard postoperative period of programming, and the stimulation voltage was kept below 3.5 V. We only observed the stimulation effect for a few minutes. Furthermore, the increment of stimulation voltage at ventral contacts was often impeded by the occurrence of side effects, such as diplopia, dizziness, or sensory complaints. Mood change was less likely to be provoked at the subthreshold stimulation intensity. Nevertheless, the determination of the hypomanic state during acute stimulation has the advantage that the neuropsychiatric change observed after switching stimulation on and off can be directly attributed to local stimulation *per se*, avoiding confounds such as medication adjustment and neuroplasticity associated with chronic stimulation. Importantly, this study only established the connection between θ oscillations of the STN region and hypomania induced by acute stimulation during the initial programming period. Whether the results predict the emotional and behavior change by chronic stimulation at HIC remains unclear. A further study correlates the intraoperative LFP at the θ range and long-termed stimulation-induced neuropsychiatric symptoms are warranted. Nevertheless, the importance of the correlation between θ activity and hypomanic induced by acute stimulation was further strengthened by previous studies, and patients with restless and mania/hypomania state elicited by acute stimulation developed psychiatric and uncontrolled behavior at chronic stimulation (Kulisevsky et al., 2002; Mallet et al., 2007; Accolla et al., 2016). It is noteworthy that hypomania was elicited in ~30% of patients with PD during the acute stimulation in our study. The prevalence was similar to a recent report that demonstrated the occurrence of impulsive control disorders and psychosis was 47 and 25%, respectively (Bove et al., 2021).

The clinical significance of this study is three-fold. The detection of θ activity intraoperatively may help inform the selection or avoidance of contacts to stimulate. Furthermore, the identification of an association between θ power and hypomania might contribute to the future establishment of DBS therapy for neuropsychiatric disorders. Finally, the real-time monitoring of θ activity in the STN may prove beneficial in closed-loop DBS, helping to avoid stimulation-induced mood disturbance.

## Conclusion

The present data suggest that intraoperative LFP recording from the DBS electrode may help predict the location of HIC after DBS in patients with Parkinson's disease. This may prove valuable, both concerning intraoperative targeting and to subsequent DBS programming.

## Data Availability Statement

The original contributions presented in the study are included in the article/supplementary material, further inquiries can be directed to the corresponding author/s.

## Ethics Statement

The studies involving human participants were reviewed and approved by Chang Gung Medical Foundation Institutional Review Board. The patients/participants provided their written informed consent to participate in this study. Written informed consent was obtained from the individual(s) for the publication of any potentially identifiable images or data included in this article.

## Author Contributions

C-CC gave the conception and established the team. Y-CC, P-LC, C-SL, and C-CC interviewed patients and collected data. Y-CC organized the database. H-TW and T-CL performed the signal analysis and the statistical analysis. P-HT and M-CY operated the deep brain stimulation. C-HY and Y-PC managed brain images. Y-CC, C-CC, H-TW, P-HT, and C-HY wrote sections of the manuscript. All authors contributed to manuscript revision, read, and approved the submitted version.

## Funding

This work was supported by the Ministry of Science and Technology, Taiwan (MOST106-2314-B-182-035, MOST108-2321-B-009-007-MY2, MOST108-2314-B-182-014-MY3, and MOST 110-2321-B-009-004) and the Chang Gung Memorial Hospital, Taiwan (CMRPG3F1423 and CMRPG3B1432).

## Conflict of Interest

The authors declare that the research was conducted in the absence of any commercial or financial relationships that could be construed as a potential conflict of interest.

## Publisher's Note

All claims expressed in this article are solely those of the authors and do not necessarily represent those of their affiliated organizations, or those of the publisher, the editors and the reviewers. Any product that may be evaluated in this article, or claim that may be made by its manufacturer, is not guaranteed or endorsed by the publisher.
